# A biomechanical matched-pair comparison of two different locking plates for tibial diaphyseal comminuted fracture: carbon fiber-reinforced poly-ether-ether-ketone (CF-PEEK) versus titanium plates

**DOI:** 10.1186/s13018-020-02096-5

**Published:** 2020-11-23

**Authors:** Kaihua Zhou, Xiaojian He, Xingguang Tao, Fugen Pan, Huilin Yang

**Affiliations:** 1 Department of Orthopedics, the First Affiliated Hospital of Soochow University, No. 899, Pinghai Road, Soochow, 215006 China; 2 Department of Orthopedics, Qingpu Branch of Zhongshan Hospital affiliated to Fudan University, No. 1158, Gongyuandong Road, Shanghai, 201700 China

**Keywords:** Tibial fracture, Internal fixation, Biomechanics, Carbon fiber-reinforced poly-ether-ether-ketone (CF-PEEK)

## Abstract

**Background:**

Several methods have been proposed to reduce plate construct stiffness and promote secondary bone healing. In this study, we explored the stiffness and strength of the new carbon fiber-reinforced poly-ether-ether-ketone (CF 50) plate compared with the titanium alloy plate (Ti6Al4V).

**Methods:**

Titanium and CF-PEEK locking plates were tested in a tibial non-osteoporotic diaphyseal comminuted fracture model to determine construct stiffness in axial compression, torsion, and bending. Subsequently, constructs were loaded until construct failure to determine construct strength.

**Results:**

Relative to the titanium locking plate, the stiffness of the CF-PEEK locking plate was 6.8% and 30.8% lower in 200 N and 700 N axial compression, respectively (*P* < 0.05), 64.9% lower in torsion (*P* < 0.05), and 48.9% lower in bending (*P* < 0.05). The strength of the CF-PEEK locking plate was only 2.6% lower under axial compression, 7.8% lower in torsion, and 4.8% lower in bending than the titanium locking plate (*P* > 0.05).

**Conclusions:**

The CF-PEEK locking plate significantly reduced axial, torsion, and bending stiffness compared with the titanium locking plate. Nonetheless, axial, torsional, and bending strength showed only a modest reduction. Considering its other advantages, which include radiolucency and artifact-free imaging, the CF-PEEK locking plate therefore deserves further clinical investigation.

Tibial diaphyseal fracture is a common clinical condition, especially in high-energy traffic accident injuries [1]. For those who need surgical intervention, the intramedullary nail is the gold standard for treatment; however, for those patients with a narrow cavity or immature epiphysis, we often use plates for effective fixation, thereby providing a good mechanical environment for fracture healing [2]. For simple fractures, we traditionally use compression plates to promote primary bone healing by absolute stability [3]. For comminuted fractures, we recommended bridge techniques with locking plates to increase callus formation [4, 5]. The locking plates allow for the use of the biological osteosynthesis (BO) fixation principle that emphasizes preservation of blood supply. However, clinical complications such as nonunion, delay union, and plate breakage are not uncommon, accounting for about 3.5–13.3% of all cases [6–12].

There are six main reasons for nonunion, delay union, and plate breakage: (1) improper selection of plate, (2) poor reduction of fracture, (3) unstable fixation, (4) improper selection of screw number and configuration, (5) excess early weight-bearing, and (6) infection. For implants, we have done a great deal of research on the length of the plate and the number and configuration of the screws [13, 14]. We also replaced stainless steel (316 L) with titanium alloy (Ti6Al4V), which has lower stiffness and induces less soft tissue reaction. Because the frequently used metal locking plates may have relatively high stiffness, they can suppress interfragmentary motion to a level insufficient for the optimal promotion of secondary bone healing [15]. Secondary bone healing is induced by interfragmentary micromotion in the millimeter range [16], with the optimal range of micromotion being 0.2–1.0 mm [17, 18]. Deficient callus formation can lead to either delayed union or nonunion with late hardware failure of the locking plate [19, 20]. Reducing the elastic modulus of the plate can also reduce the stress shielding effect. Therefore, the selection of a plate material with an appropriate elastic modulus is a problem worthy of study. With new developments in materials science, we found that plates made with carbon fiber-reinforced poly-ether-ether-ketone had a similar elastic modulus to that of the bone cortex. In the literature, there have been successful cases using CF-PEEK implants in spine, tumors, and arthroplasty [21–25]. In trauma, CF-PEEK plates have been used for proximal humeral and distal radial fractures, with clinical outcomes better than with titanium plates [26–29]. However, there has been little research done on the use of CF-PEEK plates in lower limb fractures.

The aim of this study was to test the hypothesis that the CF-PEEK locking plate can significantly reduce stiffness compared with the titanium locking plate while retaining its strength. A less-stiff yet still strong construct of the CF-PEEK locking plate could potentially enhance bone healing by promoting early interfragmentary motion.

## Materials and methods

Titanium alloy (Ti6Al4V) and CF-PEEK (CF50) locking plates were tested in a tibial non-osteoporotic diaphyseal comminuted fracture plating configuration under axial compression, torsion, and bending as in previous studies [13, 30]. The stiffness and strength of the titanium and CF-PEEK plates were determined by progressive loading of the non-osteoporotic tibial diaphysis until construct failure.

Implants and specimens

**Fig. 1:**
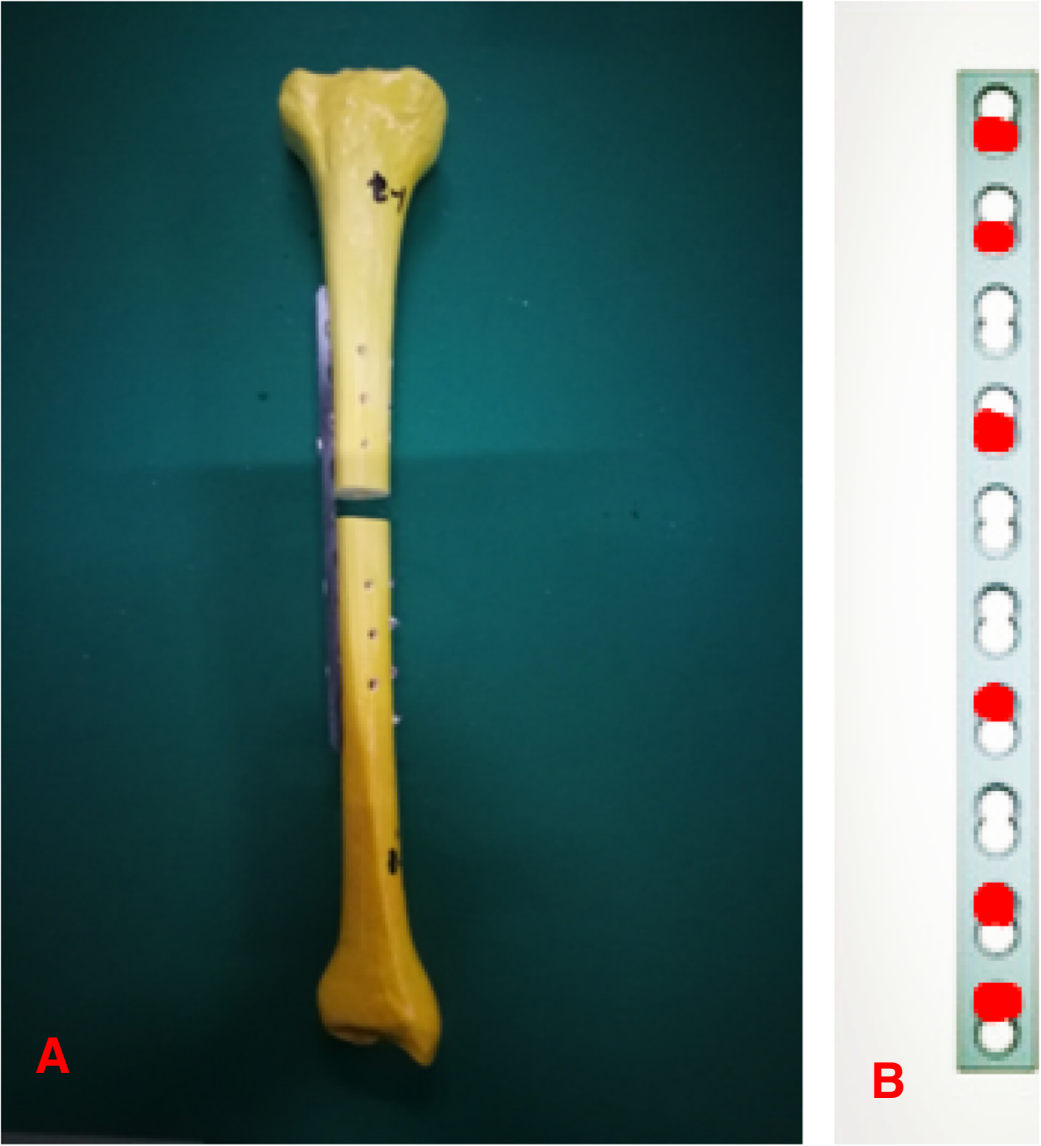
Test construct design. **a** The titanium locking plates and CF-PEEK plates were evaluated in a standard plating configuration in tibial diaphysis surrogates with a 10-mm fracture gap as comminuted fracture. **b** The plate was applied with three locking screws, which were placed in the first, second, and fourth holes in each side (red spots)

The generic titanium and CF-PEEK locking plates were designed to resemble standard broad 4.5-mm locking plates and screws. The plates were 13.5-mm wide and 181-mm long and had 10 holes with a space of 18 mm between holes. Locking screws had a 4.5-mm diameter bone thread with 1-mm pitch and a four-fluted self-tapping feature. The titanium plates were custom manufactured from surgical grade titanium alloy (Ti6Al4V) by a company specializing in the production of orthopedic implants (Watson, Jiangsu, Changzhou). CF-PEEK plates were manufactured at the same size as the titanium plates, with the PEEK material containing 50% carbon fiber. The locking screws used with the CF-PEEK plate were made of the same material as the titanium locking screws and used in the same way as in ordinary clinical use [26–29]. The titanium locking plates and CF-PEEK plates were evaluated in a standard plating configuration in tibial diaphysis surrogates with a 10-mm fracture gap as comminuted fracture [30]. For these surrogates, we used the medium-size fourth-generation composite Sawbones tibia (#3401; Pacific Research Laboratories, Vashon, WA, USA). The plate was applied with three locking screws placed in the first, second, and fourth holes in each side (Fig. 1). All screws were tightened to 4 Nm with the plate at 1 mm of elevation from the surrogate surface to simulate biological fixation with preservation of periosteal perfusion. Two holes were left empty over the fracture gap, yielding a plate span of 48 mm that bridged the gap.

### Loading

**Fig. 2:**
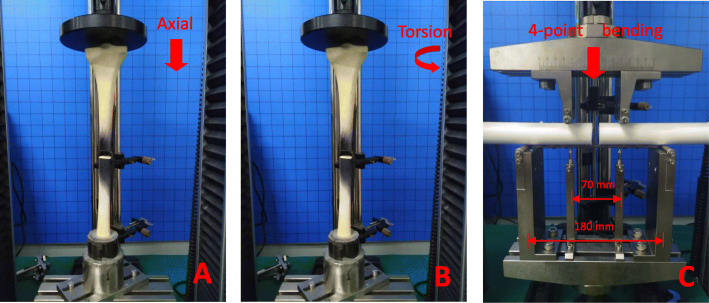
Construct stiffness and strength were evaluated under three loading conditions. **a** axial compression test, the red dot represents the location of motion-tracking sensors; **b** torsion test; **c** four point bending test in a gap-closing direction

The titanium and CF-PEEK locking plate groups were tested in axial compression, torsion, and bending with a biaxial material testing system (Instron 3365, Norwood, MA, USA) in the non-osteoporotic bone surrogate specimens (Fig. 2). A motion tracking system (Optitrack Flex13, Natural Point Inc., Corvallis, OR, USA) was used to record the data. Both groups were tested until construct failure in the axial compression, torsion, and bending loading mode, which required the use of a total of thirty-six specimens.

### Axial tests

**Fig. 3:**
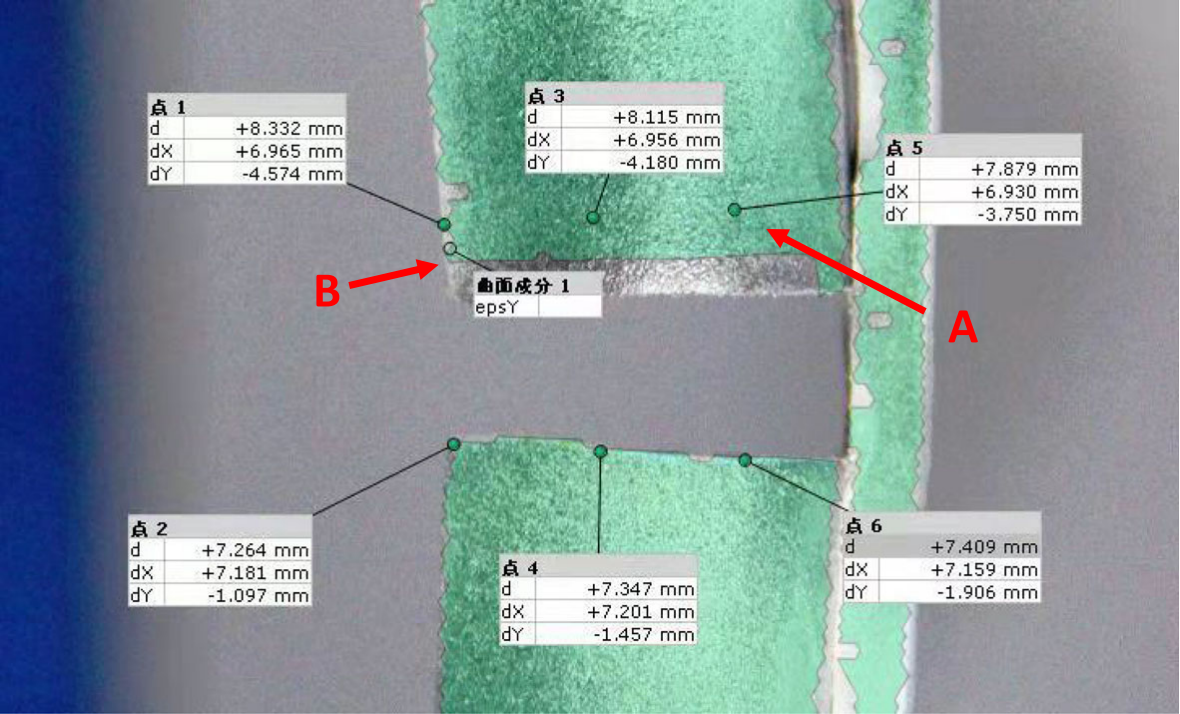
Interfragmentary motion under 200 N axial compression was also recorded at the near cortex (**a**) and far cortex (**b**)

For the axial compression test, the specimen was held vertical, while axial compression was applied by a disc at the proximal end of the specimen with the distal end embedded. The preload was 50 N, and the loading speed was 5 mm/min to 200 N and 700 N compression. Compressive stiffness was recorded, and interfragmentary motion under 200 N axial compression was also recorded at the near and far cortex (Fig. 3).

### Torsion tests

Torsion was applied around the diaphyseal axis. The two ends of the specimen were fixed in the MTS planar biaxial test system, and the machine was driven to apply torque to the specimen at a speed of 0.1/s, with the proximal end of the fracture being twisted to the outside. The rigidity and strength were recorded when the construct failed.

### Bending tests

Four-point bending was applied to generate a constant bending moment over the entire plate length. The upper and lower cylindrical supports were separated by 70 and 180 mm, respectively. The plate was fixed on the tension side to induce bending in a gap-closing mode at a speed of 5 mm/min. The rigidity and strength were recorded when the construct failed.

### Outcome evaluation

Construct stiffness was calculated from load-displacement data. Axial stiffness was calculated by dividing the axial load amplitude by the actuator displacement amplitude. Torsional stiffness was calculated by dividing the torsion amplitude by the amplitude of actuator rotation around the diaphyseal axis. Bending stiffness was calculated in terms of flexural rigidity [31]. Construct strength was defined as the peak load during progressive loading to construct failure. Construct failure was defined by either the first visible occurrence of failure of internal fixation, whether by fracture of the diaphysis through the screw hole at the plate end; plate or screw bending or breakage; fracture through the screw hole at the plate end; or a subsidence threshold at the fracture site whichever occurred first. A subsidence threshold of 10 mm in axial compression, 15° in torsion, and 10 mm in bending was deemed indicative of the onset of clinical construct failure as previous studies [31–33].

### Statistical analysis

SPSS 23.0 statistical software was used to analyze the data. One-way ANOVA was used for comparisons between multiple groups; Student’s *t* tests were used for comparisons between two groups. Differences are considered to be statistically significant when *P* values < 0.05.

## Results

### Construct stiffness (Table 1)

**Fig. 4:**
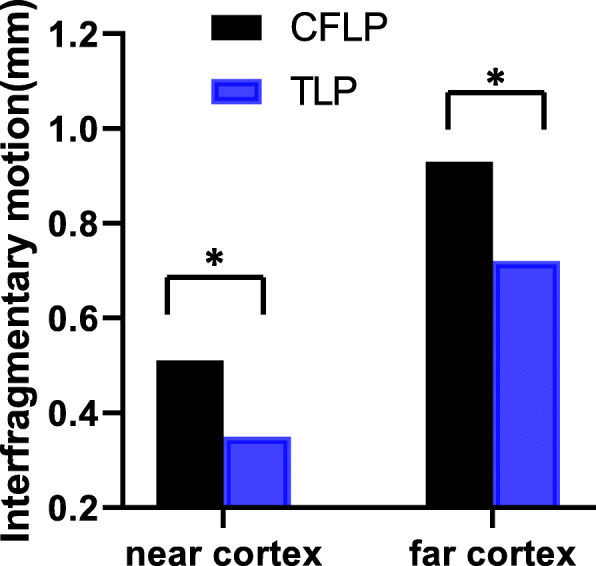
At 200 N of axial loading, the fracture site motion in the CF-PEEK locking plate (CFLP) was greater than that in the titanium locking plate (TLP). *Significant (*P* < 0.05)

With 200 N axial compression, the stiffness of the CF-PEEK plate was 6.8% lower than that of the titanium plate (169.83 ± 3.74 N/mm compared with 182.15 ± 5.53 N/mm, *P* < 0.05). The near cortex displacement of the CF-PEEK locking plate was 31.4% larger than that of the titanium plating plate (0.51 ± 0.05 mm compared with 0.35 ± 0.02 mm, *P* < 0.05). The far cortex displacement of the CF-PEEK locking plate was 22.6% larger than that of the titanium locking plate (0.93 ± 0.02 mm compared with 0.72 mm ± 0.01, *P* < 0.05) (Fig. 4).

**Table 1:** Stiffness and strength of CF-PEEK locking plate and titanium alloy locking plate

	CF-PEEK locking plate	Titanium alloy locking plate	*P* value
**Stiffness**			
Axia stiffness (N/mm)			
200 N	169.83 ± 3.74	182.15 ± 5.53	*P* < 0.05
700 N	155.05 ± 3.79	224.19 ± 16.62	*P* < 0.05
Torsional rigidity (Nm^2^/deg)	0.13 ± 0.01	0.32 ± 0.09	*P* < 0.05
Bending rigidity (Nm^2^)	0.86 ± 0.01	1.68 ± 0.01	*P* < 0.05
**Strength**			
Axial (kN)	1.79 ± 0.01	1.84 ± 0.02	*P* < 0.05
Torsion (Nm)	4.48 ± 0.05	4.86 ± 0.23	*P* < 0.05
Bending (Nm)	4.21 ± 0.01	4.42 ± 0.13	*P* < 0.05

In 700 N axial compression, the stiffness of the CF-PEEK plate was 30.8% lower than that of the titanium plate (155.05 ± 3.79 compared with 224.19 ± 16.62 N/mm, *P* < 0.05).

In the torsion test, the rigidity of the CF-PEEK plate was 59.4% lower than that of the titanium plate (0.13 ± 0.01 compared with 0.32 ± 0.09 Nm^2^/deg, *P* < 0.05).

**Fig. 5:**
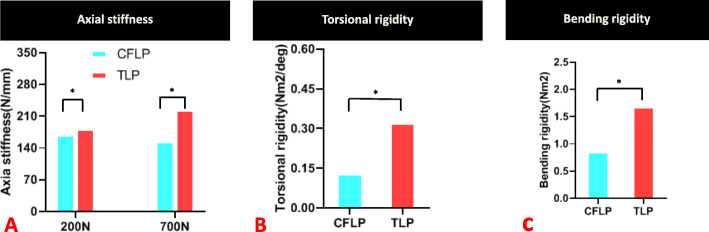
Relative to the titanium locking plate (TLP), the stiffness of the CF-PEEK locking plate (CFLP) was 6.8% and 30.8% lower in 200 N and 700 N axial compression, respectively, 59.5% lower in torsion, and 48.9% lower in bending. *Significant (*P* < 0.05)

In the bending test, the rigidity of the CF-PEEK locking plate was 48.8% lower than that of the titanium locking plate (0.86 ± 0.01 compared with 1.68 ± 0.01 Nm^2^, *P* < 0.05) (Fig. 5).

### Construct strength in the non-osteoporotic diaphysis (Table 1)

**Fig. 6:**
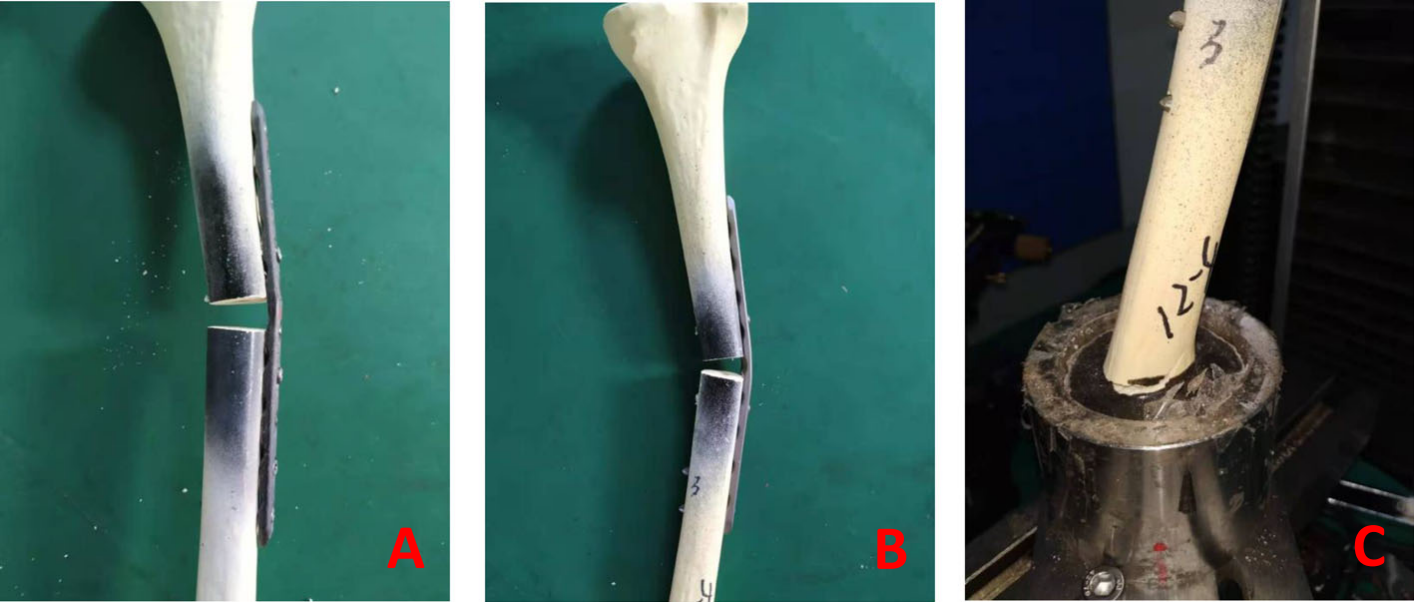
locking plate constructs failed plate: breakage (**a**); plate bending (**b**); fracture at the bone end (**c**)

**Fig. 7:**
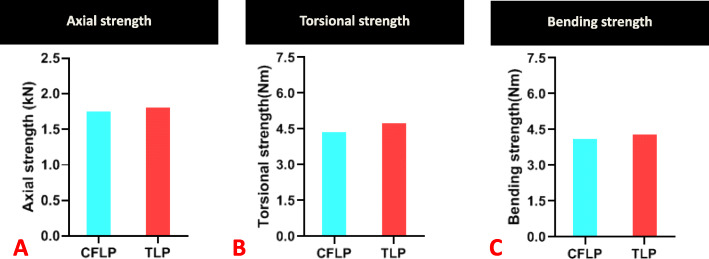
The strength of the CF-PEEK locking plate was only 2.6% lower under axial compression than the titanium locking plate (*P* < 0.05), and 7.8% lower in torsion (*P* < 0.05), and 4.8% lower in bending (*P* < 0.05)

In axial compression, the CF-PEEK locking construct was only 2.6% weaker than the titanium locking construct (1.79 ± 0.01 compared with 1.84 ± 0.02 kN, *P* > 0.05). All the constructs failed as a result of failure of internal fixation (Fig. 6). In the torsion and bending test, all the constructs failed by reaching the subsidence threshold at the fracture site. In torsion, the strength in the CF-PEEK locking construct was 7.8% weaker than that in the titanium locking construct (4.48 ± 0.05 compared with 4.86 ± 0.23 Nm, *P* > 0.05). In bending, the CF-PEEK locking construct was 4.8% weaker than the titanium locking construct (4.21 ± 0.01 compared with 4.42 ± 0.13 Nm, *P* > 0.05) (Fig. 7).

## Discussion

The results of the present study support the hypothesis that a CF-PEEK locking plate can have considerably lower stiffness than a titanium locking plate while retaining its strength. The near and far cortex displacement for both CF-PEEK and titanium locking plates was in the range of 0.2–1.0 mm micromotion, which is optimal to promote callus formation.

Fracture healing is an extremely complex biological process affected by many factors. It requires a good local mechanical environment. Fracture healing can be divided into primary and secondary healing, with secondary healing depending on callus formation. The formation of callus is affected by the range of micromotion at the fracture end. The stiffness reduction of the plate may benefit micromotion with bridge plating osteosynthesis [17, 34]. Biomechanical studies have shown that the elastic modulus of the traditional metal material is much larger than that of the bone cortex (cortex bone 18 GPa, stainless steel 200 GPa, titanium alloy 106 to 155 GPa) [32, 35], which may be a cause of nonunions [15, 34, 36]. A systematic review demonstrated a 3.5-fold increase in the rate of nonunions associated with metal locking plates as compared with intramedullary nailing [37]. Thus, we need plates that are less rigid to improve fracture healing.

There are many methods we can use to reduce the stiffness of locking plate constructs, such as increasing the plate span, plate elevation, or adjusting the screw number and configuration [38–40]. However, the strength of the locking plate is often sacrificed when its stiffness is reduced. Stoffel et al. reported that increasing the titanium plate working span could reduce the compression and torsion stiffness two-fold but also led to a 33% reduction in strength under axial compression [40]. Alternatively, increasing the plate elevation from 2 to 6 mm was reported to yield a 10% to 15% decrease in both axial and torsional rigidity [38]. However, 5 mm of plate elevation decreased construct strength in axial compression by 63% [40].

We also can choose other materials to produce the plate, such as calcium phosphate, magnesium alloys, or absorbable and degradable materials such as polylactic acid and polyhydroxyacetic acid to replace the traditional metal materials [41]. Although animal experiments showed that the stiffness and stress shielding can thereby be significantly reduced, the strength of the plate became poor. In addition, the degradation rate of the absorbable plate is difficult to control, especially in the internal fixation of long bone fractures [42, 43]. Over the past years, poly-ether-ether-ketone (PEEK) has seen increasing use in medical materials. Through technical innovation, we embedded carbon fibers (CF) into PEEK in different directions to form a new composite material, CF-PEEK. The elastic modulus of CF-PEEK is smaller than that of metal plates and closer to that of cortical bone. The stiffness and strength are significantly improved and can fix the fracture firmly without losing the original advantages of PEEK.

In the axial compression, torsion, and bending tests, the stiffness in the CF-PEEK locking plate decreased compared with the titanium locking plate. An animal study showed that a decrease in fixation stiffness caused a significant increase in the rate of fracture healing in a sheep model [16]. Research shows that micromotion caused by axial compression load is beneficial to fracture healing, and the micromotion distance of 0.2–1.0 mm is appropriate. If it exceeds 2 mm, it may have a negative effect on fracture healing because excessive interfragmentary motion can lead to hypertrophic callus formation and nonunion [17, 18]. Under axial compression of 200 N as the partial weight-bearing condition, both near and far cortex micromotion in the CF-PEEK plate is larger than that of titanium plating construct, but within the optimal range.

The titanium alloy locking plate is the most commonly used plate for lower limb fractures in the clinic. Meanwhile, the results of this study showed that the strength of the CF-PEEK locking plate is no less than that of the traditional titanium alloy locking plate. This indicated that the new material plate is safe in the treatment of tibial shaft comminuted fracture in theory, although further research is needed.

There are many other advantages of the CF-PEEK material that prompt us to study it further, including: (1) good biocompatibility to reduce allergic reaction to the plate; (2) radiolucency and artifact-free imaging, so we can better observe the reduction quality of the fracture during the operation and the formation of callus after operation; (3) no galvanic corrosion, cold welding, or metal ion release, which can reduce the difficulty of removing the plate; and (4) increased accuracy of radiotherapy dosing in cancer patients, with Christoph et al. reporting that CF/PEEK implants lead to a more reliable and more effective delivery of radiation dose to an osseous target [44]. Thus, the CF-PEEK plate is a more ideal material for bone plates both in biomechanics and in other attributes.

Our study has two potential limitations. First, we investigated axial loading, torsion, and bending individually. Though this enables us to understand the benefits and weaknesses of CF-PEEK locking constructs, loading in clinical situations will be some combination of the above forces with more complex biomechanics. Second, bone quality will also affect the micromotion of the fracture ends after plate fixation. Thus far, we have only carried out biomechanical tests on normal bone; we will conduct relevant tests on osteoporotic bone in the future.

In conclusion, CF-PEEK plates appear to offer an attractive alternative to reduce the stiffness while retaining the strength of bridge plating constructs when interfragmentary motion is desired to promote secondary bone healing. Despite the theoretical benefits of CF-PEEK locking plates, future in vivo studies will be required to evaluate whether CF-PEEK locking plates can better promote formation and maturation of a fracture callus than metal plates.

## Data Availability

The datasets used and/or analyzed during the current study are available from the corresponding author on reasonable request.

## Abbreviations

CF: Carbon fiber
CF-PEEK plate: Carbon fiber reinforced poly-ether-ether-ketone composite plate
PEEK: Poly-ether-ether-ketone
AO: Arbeitsgemeinschaftfür Osteosynthesefragen
CFLP: Carbon fiber reinforced poly-ether-ether-ketone composite locking plate
TLP: Titanium alloy locking plate
